# Vitamin B12 analogues from gut microbes and diet differentially impact commensal propionate producers of the human gut

**DOI:** 10.3389/fnut.2024.1360199

**Published:** 2024-02-08

**Authors:** Palni Kundra, Anna Greppi, Monica Duppenthaler, Serafina Plüss, Benoit Pugin, Christophe Lacroix, Annelies Geirnaert

**Affiliations:** Laboratory of Food Biotechnology, Institute of Food, Nutrition and Health, Department of Health Sciences and Technology, Zurich, Switzerland

**Keywords:** cobalamin, pseudo-cobalamin, B12-analogues, B12-prototrophs, propionate, gut microbiota, *Akkermansia muciniphila*, *Bacteroides thetaiotaomicron*

## Abstract

To produce the health-associated metabolite propionate, gut microbes require vitamin B12 as a cofactor to convert succinate to propionate. B12 is sourced in the human gut from the unabsorbed dietary fraction and *in situ* microbial production. However, experimental data for B12 production by gut microbes is scarce, especially on their produced B12-analogues. Further, the promotion of propionate production by microbially-produced and dietary B12 is not yet fully understood. Here, we demonstrated B12 production in 6 out of 8 *in silico* predicted B12-producing bacteria from the human gut. Next, we showed *in vitro* that B12 produced by *Blautia hydrogenotrophica*, *Marvinbryantia formatexigens,* and *Blautia producta* promoted succinate to propionate conversion of two prevalent B12-auxotrophic gut bacteria, *Akkermansia muciniphila* and *Bacteroides thetaiotaomicron*. Finally, we examined the propiogenic effect of commercially available B12-analogues present in the human diet (cyano-B12, adenosyl-B12 and hydroxy-B12) at two doses. The low dose resulted in partial conversion of succinate to propionate for *A. muciniphila* when grown with adenosyl-B12 (14.6 ± 2.4 mM succinate and 18.7 ± 0.6 mM propionate) and hydroxy-B12 (13.0 ± 1.1 mM and 21.9 ± 1.2 mM), in comparison to cyano-B12 (0.7 ± 0.1 mM and 34.1 ± 0.1 mM). Higher doses of adenosyl-B12 and hydroxy-B12 resulted in significantly more conversion of succinate to propionate in both propionate-producing species, compared to the low dose. B12 analogues have different potential to impact the propionate metabolism of prevalent propionate producers in the gut. These results could contribute to strategies for managing gut disorders associated with decreased propionate production.

## 1 Introduction

Propionate, one of the short-chain fatty acids produced by certain gut microbes during carbohydrate fermentation, has several health benefits extending beyond the gut (1, 2). For example, propionate has been shown to prevent body weight gain, reduce intra-abdominal fat accumulation and hence obesity, and decrease intrahepatocellular lipid content, because it affects satiety and energy homeostasis by several complementary mechanisms (3, 4). It can also reduce low density and total cholesterol levels and possibly attenuate atherosclerosis as demonstrated in both human and mice studies (5). It may also prevent development of cancer in other parts of the body (6, 7). Additionally, an *in vitro* study showed that a propionate-producing consortium of gut bacterial strains can restore antibiotic-induced gut microbiota dysbiosis (8). Because of these benefits, many studies have been conducted to investigate the potential benefits of selectively increasing propionate production in the colon through consumption of non-digestible carbohydrates in the diet, with the aim of promoting overall health (9–12).

Propionate is primarily produced by gut bacteria through three different pathways: the succinate, the acrylate, and the propanediol pathway, of which the succinate pathway is the most prevalent in the human gut microbiota (13). For production of propionate via the succinate and the propanediol pathways, vitamin B12 (B12) is required as a cofactor for methylmalonyl-CoA mutase and glycerol/diol dehydratases, respectively (14). *In vitro* studies have shown that B12 supplementation can enhance propionate production by single microbes such as *Akkermansia muciniphila*, and also by complex human gut microbiota in fecal batch fermentation (15, 16). However, the effect of exogenous B12 on propionate production is not yet fully understood, as it varies depending on the donor microbiota (16).

B12 is a group of water-soluble B-vitamins, referred to as B12 analogues, that are required by all forms of life but are exclusively produced by prokaryotes (17). Structurally, B12 contains a corrin ring attached to a central cobalt molecule with upper and lower axial ligands and belongs therefore to the cobamides that are exclusively produced by prokaryotes (18). The upper axial ligand can vary between cyano-, methyl-, adenosyl-, or hydroxy-B12, while the lower ligand contains either dimethyl-benzimidazole or adenine (19). When the lower ligand is adenine, the resulting B12 is called pseudo-cobalamin (pseudo-B12). This form is not suitable for human cells as it has a low bioavailability and biological activity, but this form is a major form of B12 in human feces (18, 20–22).

B12 present in the gut environment can either originate from unabsorbed dietary sources or from *in situ* gut microbial production. Dietary sources can contain different B12 analogues depending on the food product consumed, ranging from the artificial stable cyano-B12 used for food fortification to the adenosyl-, methyl-, or hydroxy-B12 forms naturally present in food products such as meat, fish and eggs (23, 24). Furthermore, metagenomic studies have revealed the presence of B12-producing microbes (prototrophs) in the gut (14, 25, 26). Similarly, microbially-produced B12 can also have structurally diverse B12 forms including pseudo-B12 (14, 15, 18). One study reported that pseudo-B12 produced by the gut bacterium *Eubacterium hallii* can promote propionate production by *A. muciniphila* (15). Nevertheless, further experimental data on B12 production by single gut microbes is lacking, and very few studies have reported B12 exchange and utilization by the other gut microbes (15).

Propionate-producing species exhibit specificity in utilizing different analogues of B12. For instance, the B12 auxotroph *A. muciniphila*, which produces propionate via the succinate pathway, has the ability to convert (remodel) any analogue of B12 to pseudo-B12 (27). On the other hand, *Bacteroides thetaiotaomicron,* another prevalent and abundant propionate-producing and B12 auxotroph species in the human gut, encodes multiple B12 transporters that selectively capture different B12-like small molecules, called corrinoids (28). However, the effect of different microbially-produced and commercially available B12 analogues on propionate production is still not known.

To address these knowledge gaps, this study analyzed *in vitro* B12 production capacity by gut bacterial strains predicted as B12 prototrophs, and quantified the B12 analogue produced. Next, this study investigated if the gut microbially-produced B12 promotes growth and propionate production by two common propionate-producing and B12 auxotrophic gut bacteria: *A. muciniphila* and *B. thetaiotaomicron*. Further, it was investigated whether the B12 analogues (cyano-B12, adenosyl-B12 and hydroxy-B12) or their dose impact auxotrophic growth and propionate production. Ultimately, this study aimed to better understand the role and relevance of gut microbially-produced and dietary B12 analogues on propionate metabolism in the human gut.

## 2 Materials and methods

### 2.1 Bacterial strains and cultivation conditions

All strains were acquired from the German Collection of Microorganisms and Cell Culture GmbH (DSMZ, Braunschweig, Germany). Strains were stored at −80°C in 25% (v/v) anaerobic glycerol stocks, and were routinely cultivated in anaerobic gut basal medium (BM), consisting of (per liter): 20 g tryptone (Chemie Brunschwig AG, Basel, Switzerland), 4 g L-threonine, 0.5 mg resazurin (redox indicator), 4 g NaHCO_3_, 1 g L-cysteine•HCl, 0.4 g KH_2_PO_4_ (VWR International AG, Dietikon, Switzerland), 0.53 g Na_2_HPO_4_, 0.3 g NH_4_Cl, 0.3 g NaCl, 0.1 g MgCl_2_•6H_2_O, 0.11 g CaCl_2_, 1 mL of acid trace element stock solution (per liter: 50 mM HCl (VWR International AG), 1 mM H_3_BO_3_, 0.5 mM MnCl_2_, 7.5 mM FeCl_2_, 0.5 mM CoCl_2_, 0.1 mM NiCl_2_, and 0.5 mM ZnCl_2_), and 1 mL of alkaline trace element stock solution (per liter: 10 mM NaOH, 0.1 mM Na_2_SeO_3_, 0.1 mM Na_2_WO_4_, and 0.1 mM Na2MoO_4_) (29). The medium was supplemented with the following vitamins at final concentration (per liter): 20 μg biotin, 20 μg nicotinic acid, 10 μg p-aminobenzoic acid, 20 μg thiamine, 20 μg pantothenate, 50 μg pyridoxamine, 10 μg cyano-B12 (added to precultures but not added to experiment medium), 20 μg folic acid, 10 μg riboflavin, 5 μg lipoic acid, 10 μg menadione and 0.75 μg phylloquinone (30). The carbohydrate substrates added to the BM medium were glucose (27.7 mM) or a mix of glucose (13.9 mM) and N-acetyl-D-Glucosamine (GlucNAC, 12.4 mM) for *Akkermansia muciniphila* DSM 22959, *Bacteroides thetaiotaomicron* DSM 2079 (only during *in vitro* screening for B12 production), and *Ruminococcus gnavus* ATCC 29149. Next, the BM medium was supplemented with either acetate (33 mM; BMA; Chemie Brunschwig AG) for *Anaerostipes caccae* DSM 14662, *Blautia hansenii* DSM 20583, *Blautia producta* DSM 14466, *Blautia obeum* DSM 25238, *Ruminococcus gauvreauii* DSM 19829, *Ruminococcus gnavus* ATCC 29149, *Anaerostipes hadrus* DSM 3319, *Akkermansia muciniphila* DSM 22959, *Bacteroides thetaiotaomicron* DSM 2079, *Faecalibacterium prausnitzii* A2-165 or formate (50 mM; BMF) for *Blautia hydrogenotrophica* DSM 10507 T and *Marvinbryantia formatexigens* DSM 14469. Anaerobic media preparation and cultivation were conducted as previously described in Hungate tubes (N_2_ headspace) and in 96-deep well plates (31, 32). All chemicals were ordered from Sigma-Aldrich Chemie GmbH (Buchs, Switzerland) unless otherwise stated.

Because low propionate production was detected in experiments with *B. thetaiotaomicron* in BMA medium, the experiments were also performed in a chemically defined medium (CDM) that was previously developed to cultivate gut anaerobes (33). Compared to BMA, CDM had a different nitrogen source composition and contained 10 g/L of vitamin-free casein acid hydrolysate (Sigma-Aldrich Chemie GmbH), instead of 20 g/L of tryptone in BMA. Additionally, CDM contained a mixture of amino acids, whereas BMA contained L-threonine (33). However, both CDM and BMA contained glucose as carbohydrate source (27.7 mM) for cultivation of *B. thetaiotaomicron*.

Strains were reactivated from glycerol stocks and sub-cultured at least twice before the start of the experiments in Hungate tubes (N_2_ headspace) at 2% inoculum and were incubated for 48 h at 37°C in independent triplicates. Before every experiment, samples were taken from the inoculum for gram staining to confirm the purity of the cultures.

### 2.2 *In vitro* screening of gut bacterial strains for B12 production

For *in vitro* screening of B12 production, a total of 12 gut bacterial strains (8 potential B12 producers and 4 non-producers) were chosen based on their predicted genetic ability to produce B12 (25, 26). Pre-cultures were inoculated directly in their respective medium without added B12 in 96-deep well plates (2.2 mL, Milian SA, Vernier, Switzerland) sealed with breathable seals (Sigma-Aldrich Chemie GmbH). Plates were incubated for 24 h at 37°C in an anaerobic chamber (10% CO_2_, 5% H_2_, and 85% N_2_; Coy Laboratories, Grass Lake, MI, United States). The screening was initiated by inoculating 2% (v/v) of pre-cultures to 2 mL of fresh medium without B12 for 48 h at 37°C in biological triplicates. After incubation, 100 μL of sample was transferred to another 96-microtiter plate (300 μL, Milian SA), and optical density was measured at 600 nm using a plate reader (OD_600_, Tecan Trading, Männedorf, Switzerland). For quantification of B12 and total bacteria, two 0.8 mL aliquots were transferred to 96 deep-well plates (Milian SA) and centrifuged for 10 min (5,000 x *g*, 4°C) (Sorvall LYNX 6000 Centrifuge, Thermo Scientific). The supernatant was transferred to a new 96 deep-well plate. Plates with pellet and supernatant were stored at −80°C until further analysis.

### 2.3 Preparation of inactivated bacterial (IB) preparations

Use of microbially-produced B12 by the propionate-producing B12 auxotrophic strains *A. muciniphila* and *B. thetaiotaomicron* was investigated by cultivation in medium containing IB preparations produced by B12 prototrophs (*B. producta*, *M. formatexigens*, and *B. hydrogenotrophica*) and a non-producer (*F. prausnitzii*). Therefore, IB preparations were prepared by culturing B12 prototrophic strains in anaerobic serum flasks (100 mL; N_2_ headspace; Infochroma AG, Goldau, Switzerland) containing 50 mL BMF (*B. hydrogenotrophica* and *M. formatexigens*) or BMA (*B. producta* and *F. prausnitzii*) without B12 for 48 h at 37°C. The culture suspension was heat-treated (100°C for 30 min) to release intracellular B12, with vortexing every 10 min to facilitate extraction (34). Afterwards, the samples were placed on ice, centrifuged at 12,000 x *g* for 10 min at 4°C, and supernatants were then separated from cell debris. The resulting IB preparations were filter-sterilized (0.2 μm; Sartorius Minisart-Plus, VWR International AG) and stored in 8 mL aliquots at −80°C in autoclaved Hungate tubes pre-flushed with nitrogen. A sample of approximately 0.8 mL was taken to quantify the B12 present in the IB preparations and confirm B12 prototrophy of the tested strains.

### 2.4 B12 auxotrophs growth experiments with IB preparations

The growth and propionate production of the two B12 auxotrophic strains, *A. muciniphila* and *B. thetaiotaomicron*, was assessed in a sterile 96 deep-well plate (Milian SA) supplemented with and without different B12 sources (cyano-B12 or IB preparations of B12 prototrophs). Two times concentrated BMA medium (0.6 mL) was supplemented with an equal volume (0.6 mL) of the sterile IB preparations of the confirmed B12 prototrophs (*B. producta*, *M. formatexigens*, and *B. hydrogenotrophica*) and *F. prausnitzii* (used as negative control). In addition, the B12 auxotrophic strains were cultivated in BMA without or with cyano-B12 (10 ng/mL cyanocobalamin).

Pure cultures of two propionate-producing strains, *A. muciniphila* (glucose and GlucNAC as C-sources) and *B. thetaiotaomicron* (glucose as C-source), in monoculture were inoculated after cell harvesting and washing. Briefly, the OD_600_ of the grown cultures was measured and normalized to an OD_600_ equal to 1 by dilution with anaerobic phosphate buffered saline (PBS; composition in g/l: K_2_HPO_4_ (Sigma-Aldrich Chemie GmbH), 8.8; KH_2_PO_4_ (VWR International AG), 6.8; sodium chloride (Sigma-Aldrich Chemie GmbH), 8; L-cysteine•HCl (Sigma-Aldrich Chemie GmbH), 1 and resazurin stock solution, 0.0005). The normalized cell suspension was washed three times under anaerobic conditions with anaerobic PBS to prevent carryover of B12 from the initial inoculum culture (5,000 x *g* for 10 min). After inoculation at 2.5% (v/v), the plate was sealed with a breathable seal (Sigma-Aldrich Chemie GmbH) and incubated at 37°C for 72 h under anaerobic conditions in an anaerobic chamber to maintain anaerobiosis. A sample (200 μL) was taken for OD_600_ in the plate reader (BioTek, PowerWave XS, BioTek Instruments, Inc., Vermont, United States). The remaining volumes were then centrifuged for 20 min (5,000 x *g*, 4°C) (Sorvall LYNX 6000 Centrifuge, Thermo Scientific), and the supernatants were stored at −20°C until further analysis. All conditions were tested in three biological replicates.

### 2.5 B12 auxotrophs growth experiments with different analogues of B12

Growth and metabolite production of *A. muciniphila* and *B. thetaiotaomicron* were tested with supplementation of different analogues of B12 (cyano-, adenosyl- and hydroxy-B12; all from Sigma-Aldrich Chemie GmbH) and without B12 in 96 deep-well plates (Milian SA). These analogues of B12 are used in diet and dietary supplements and also found in human feces (21, 35). B12 forms were tested at two doses, 10 μg/L (1x) and a 20-fold higher dose of 200 μg/L (20x), which are, respectively, two times and 40 times higher than in the medium used for human colon microbiota cultivation (16, 36). These concentrations were based on suggested concentrations for cultivating single gut microbes (10 μg/L) (33). In comparison, under normal dietary conditions of 4.5 μg/day, approximately 3.75 μg/L of B12 is expected to reach the colon from dietary sources, which increases to 2,500 μg/L when taking supplements equivalent to a single dose of 1,500 μg/day per tablet (16). Strains were cultivated in monocultures in BMA and inoculated after cell washing as described above. After inoculation, the plate was sealed and incubated at 37°C for 72 h under anaerobic conditions in an anaerobic chamber. The OD_600_ was measured with the plate reader, and supernatant was collected and stored as presented above. All conditions were tested in three biological replicates.

### 2.6 B12 Quantification by ultra high-performance liquid chromatography-diode array detector (UHPLC-DAD)

B12 was quantified as pseudo-B12 (lower ligand adenine) and cyano-B12 (lower ligand dimethyl-benzimidazole). Extraction was performed as described previously (34), with addition of potassium cyanide (KCN) to the samples to convert all B12 forms into the stable form with a cyano group as upper ligand. Both intracellular and extracellular B12 were measured separately for each culture. For quantification of IB preparations and extracellular samples, 0.8 mL extraction buffer (8.3 mM NaOH and 20.7 mM acetic acid) and 8 μL of 1% KCN solution were added to 0.8 mL sample and vortexed for 10 s. For intracellular quantification, pellets were resuspended in 0.8 mL extraction buffer, 8 μL 1% KCN solution was added, and samples were vortexed for 10 s. Samples were then placed in a water bath at 100°C for 30 min, afterwards cooled on ice for 10 min, and centrifuged for 20 min at 4°C (10,000 x *g*). Supernatant was filtered through a 0.20 μm nylon membrane filter (VWR International AG) into brown vials (BGB Analytik AG). All analyses were carried out under red or low light conditions to avoid B12 degradation during sample processing.

B12 quantification was performed on a Thermo Scientific Vanquish UHPLC equipped with a Diode Array Detector (DAD), using an Acquity HSS T3 column (2.1 × 1,000 mm, 1.8 μm; Waters AG, Baden-Dättwil, Switzerland) connected to ACQUITY UPLC HSS T3 VanGuard Pre-column (100 Å, 1.8 μm, 2.1 mm x 5 mm; Waters AG). The detection was conducted at 362 nm. The autosampler was set at 12°C and the column was operated at 30°C, and 20 μL of sample was injected. For separation, acetonitrile (A) and MilliQ water (B) containing 0.1% formic acid was used as mobile phase, with a gradient flow (0.5 mL/min): 0–0.5 min [A 5% and B 95%]; 0.5–5 min [A 40% and B 60%]; 5–6 min [A 40% and B 60%]; 6–10 min [A 95% and B 5%]; 10–11 min [A 5% and B 95%]; 11–15 min [A 5% and B 95%]. The calibration curve was obtained by measuring cyano-B12, adenosyl-B12, hydroxy-B12 and methyl-B12 standards (Sigma-Aldrich Chemie GmbH) in the 60–4,000 ng/mL range, while pseudo-B12 was prepared as an extract from spirulina tablets, (37) with standards ranging from 30–1,100 ng/mL. Based on linearity, the limit of detection was 30 ng/mL. The B12 specific yield was expressed as ng/10^9^ cells, with the total cell numbers measured with quantitative PCR, as presented below.

### 2.7 Organic acid analysis by high performance liquid chromatography with refractive index detector (HPLC-RI)

Organic acids, i.e., SCFA (acetate, propionate, butyrate, and valerate), intermediate metabolites (lactate, succinate, and formate), and branched-chain fatty acids (BCFA; isobutyrate and isovalerate) were measured by HPLC-RI as reported previously (16). Briefly, 200–300 μL of supernatant was filtered through a 0.20 μm nylon membrane filter (VWR International AG) into a vial (BGB Analytik AG, Boeckten, Switzerland).

Samples were analyzed using a HPLC (Hitachi LaChrom, Merck, Dietikon, Switzerland) equipped with a precolumn SecurityGuard Cartridges Carbo-H (4 × 3.0 mm; Phenomenex Helvetia GmbH, Basel, Switzerland) connected to a Rezex ROA-Organic Acid H+ column (300 × 7.8 mm; Phenomenex Helvetia GmbH) and a refractive index detector. Samples (40 μL injection volume) were eluted at 40°C under isocratic conditions with 10 mM H_2_SO_4_ at a flow rate of 0.4 mL/min. All metabolites were quantified using external standards (Sigma-Aldrich Chemie GmbH for all, except valeric acid and formic acid from VWR International AG).

### 2.8 DNA extraction

DNA was extracted from 0.6 to 1 mL of fermentation sample using the FastDNA Spin Kit for Soil (MP Biomedicals, Illkirch-Graffenstaden, France), following the manufacturer’s protocol as reported previously (16). Briefly, samples were filled into 2 mL tubes containing Lysing Matrix E and homogenized (40 s, 6.0 m/s) in a FastPrep® instrument in the presence of lysis buffer solution and sodium phosphate buffer. Following lysis, samples were centrifuged, and DNA was purified from the supernatant with a silica-based filter in a final volume of 100 μL. DNA was quantified using a Nanodrop® ND-1000 Spectrophotometer and stored at −20°C until further analysis.

### 2.9 Quantification of total bacteria

Quantitative PCR was used to quantify total bacteria using primers Eub_338F (5′-ACTCCTACGGGAGGCAGCAG-3′) and Eub_518R (5′-ATTACCGCGGCTGCTGG-3′) as reported previously (16). All reactions consisted of 5 μL of 2X SensiFAST SYBR No-ROX Kit master mix (Meridian Bioscience, Cincinnati, OH, United States), 0.5 μL of each forward and reverse primer (final concentration of 0.5 μM each), 1 μL of DNA and 3 μL of nuclease-free water. Reactions per sample were performed in triplicate in 96-well plates using a LightCycler 480 qPCR II system (Roche Diagnostics, Rotkreuz, Switzerland) and a two-step program consisting of 3 min of initial denaturation at 95°C, followed by 40 cycles of 95°C for 5 s and 65°C for 30 s. For quantification, a 10-fold dilution series of standards containing a linearized plasmid with the *Escherichia coli* 16S rRNA gene was included in each run. PCR efficiency (%) was calculated from the slope of the standard curve of each qPCR assay. Assays with an efficiency of 80–110% (slope of 3.2–3.9) were included in the data analysis. The qPCR gene copy number was transformed to cell concentration by normalizing for the median of 16S rRNA gene copy number per bacterium based on the Ribosomal RNA Database when available; otherwise, the average of five 16S rRNA gene copies was used (38).

### 2.10 Statistical analysis

Statistical analyses for OD_600_ and metabolite data was done using GraphPad Prism v.9.2.0. One-way ANOVA with Tukey’s test to correct for multiple comparison was performed to detect statistical differences (*p* < 0.05). Normality of residuals and homogeneity of variance of the data were validated using the Shapiro–Wilk test and Brown-Forsythe test, respectively.

## 3 Results

### 3.1 B12 Production by human gut bacterial strains

Twelve gut microbial strains were selected based on their predicted genetic ability to produce B12 (Table 1, 8 predicted B12 producers and 4 predicted non-producers (25, 26)) and were screened *in vitro* for B12 production. The strains were grown in medium without B12, and intra- and extra-cellular B12 and pseudo-B12 production were quantified after 48 h incubation after its conversion to the stable analogue with cyano group as upper ligand.

**Table 1:** Specific intracellular and extracellular B12 production (expressed as ng/10^9^ cells) of human gut bacterial strains after 48 h incubation under anaerobic condition at 37° C.

Strain	Predicted producer (+)/ non-producer (−)*	Intracellular pseudo-B12 (ng/10^9^ cells)	Intracellular B12 (ng/10^9^ cells)	Extracellular pseudo-B12 (ng/10^9^ cells)	Extracellular B12 (ng/10^9^ cells)	Total B12 (ng/10^9^ cells)	Log total bacteria (cells/ml)
*Anaerostipes caccae* **DSM 14662**	+	ND	39.5 ± 12.7	ND	ND	39.5 ± 12.7	9.8 ± 0.1
*Blautia hansenii* DSM 20583	+	ND	ND	ND	ND	ND	8.7 ± 0.1
*Blautia hydrogenotrophica* **DSM 10507 T**	+	30.3 ± 11.6	61.4 ± 44.3	ND	ND	91.7 ± 33.3	9.1 ± 0.3
*Blautia producta* **DSM 14466**	+	31.4 ± 5.5	ND	252.1 ± 7.5	ND	283.6 ± 2.0	9.2 ± 0.1
*Blautia obeum* **DSM 25238**	+	40.6 ± 5.4	55.01 ± 6.6	ND	ND	95.6 ± 12.2	9.4 ± 0.01
*Marvinbryantia formatexigens* **DSM 14469**	+	243.4 ± 23.2	74.2 ± 23.2	ND	ND	317.6 ± 46.4	9.0 ± 0.2
*Ruminococcus gauvreauii* **DSM 19829**	+	266.5 and 129.8	35.1 and 24.9	ND and 180.8	ND	301.6 and 335.5	9.1 and 9.5
*Ruminococcus gnavus* ATCC 29149	+	ND	ND	ND	ND	ND	9.3 ± 0.1
*Anaerostipes hadrus* DSM 3319	−	ND	ND	ND	ND	ND	8.9 ± 0.1
*Akkermansia muciniphila* DSM 22959	−	ND	ND	ND	ND	ND	9.1 ± 0.1
*Bacteroides thetaiotaomicron* DSM 2079	−	ND	ND	ND	ND	ND	9.3 ± 0.01
*Faecalibacterium prausnitzii* A2-165	−	ND	ND	ND	ND	ND	8.4 ± 0.2

B12 was measured as pseudo-cyanocobalamin (pseudo-B12) and cyanocobalamin (B12) using UHPLC-DAD. Strains that show B12 production are marked in bold. Total B12 represents the sum of all fractions (intra- and extracellular of B12 and pseudo-B12). Total bacteria were measured using qPCR. Data are means and standard deviations of three biological replicates except for *Ruminococcus gauvreauii* (*n* = 2).

*References: (14, 24, 25, 39). ND: not detected, limit of detection of 30 ng/mL.

All strains grew in their respective media as confirmed by an increase of OD_600_ (ΔOD_600_ ranging between 0.2 ± 0.02 to 0.8 ± 0.1; Supplementary Figure S1) and total bacteria qPCR results (between 8.4 ± 0.3 to 9.8 ± 0.1 log total bacterial cells/ml; Table 1). B12 was detected with Ultra High-Performance Liquid Chromatography-Diode Array Detector (UHPLC-DAD; limit of detection = 30 ng/mL) in 6 of the 8 predicted B12-producing strains, mainly in the intracellular fraction (Table 1; see also Supplementary Figures S2, S3). Both pseudo-B12 and B12 specific productions were quantified in the intracellular fraction of *M. formatexigens* (243.4 ± 23.2 ng/10^9^ cells and 74.2 ± 23.2 ng/10^9^ cells), as well as for both replicates of the *R. gauvreauii* strain (average 198.1 ng/10^9^ cells and 30.0 ng/10^9^ cells) and in two *Blautia* strains, *B. hydrogenotrophica* (30.3 ± 11.6 ng/10^9^ cells and 61.4 ± 44.3 ng/10^9^ cells) and *B. obeum* (40.6 ± 5.4 ng/10^9^ cells and 55.0 ± 6.6 ng/10^9^ cells). Only B12 was detected in intracellular samples for *A. caccae* (39.5 ± 12.7 ng/10^9^ cells). Notably, *B. producta* produced only pseudo-B12, which was detected in both intracellular (31.4 ± 5.5 ng/10^9^ cells) and extracellular (252.2 ± 7.5 ng/10^9^ cells) fractions (Table 1). Furthermore, for the predicted non-producing strains *F. prausnitzii*, *A. hadrus*, *A. muciniphila* and *B. thetaiotaomicron*, no analogue of B12 was detected. Overall, all the predicted prototrophs, except for *R. gnavus* and *B. hansenii,* produced detectable levels of B12 during batch cultivation.

### 3.2 B12 produced by B12 prototrophic strains is used by B12 auxotrophic strains for propionate production

The next objective was to investigate whether the B12 produced by certain strains can be utilized by B12 auxotrophic propionate-producing strains for promoting conversion of succinate to propionate.

Two propionate-producing model gut strains, *A. muciniphila* and *B. thetaiotaomicron*, were chosen because of their prevalence in the human gut. These strains were cultivated in the presence of inactivated bacterial (IB) preparations obtained from one of the three selected B12 prototrophic strains (*B. hydrogenotrophica, B. producta,* and *M. formatexigens*) or of the negative control strain (*F.prausnitzii*), as well as in anaerobic gut basal medium with acetate (BMA) containing 10 ng cyano-B12/ml or no B12. B12 was detected in all IB preparations as pseudo-B12 (Supplementary Table S1). Possible degradation of certain B12 analogues during IB preparation (treated without added KCN) may explain that they were below detection while they were detected in the heat-treated cultures (Table 1). The IB preparations were added at the equivolume to the two times concentrated cultivation medium resulting in 44.2, 75.1, and 65.3 ng pseudo-B12/ml at the start of incubations for the conditions with IB from *B. hydrogenotrophica, B. producta,* and *M. formatexigens,* respectively. The SCFA concentrations derived from the different IB preparations were recorded prior to incubation (Supplementary Figure S4).

**Figure 1:**
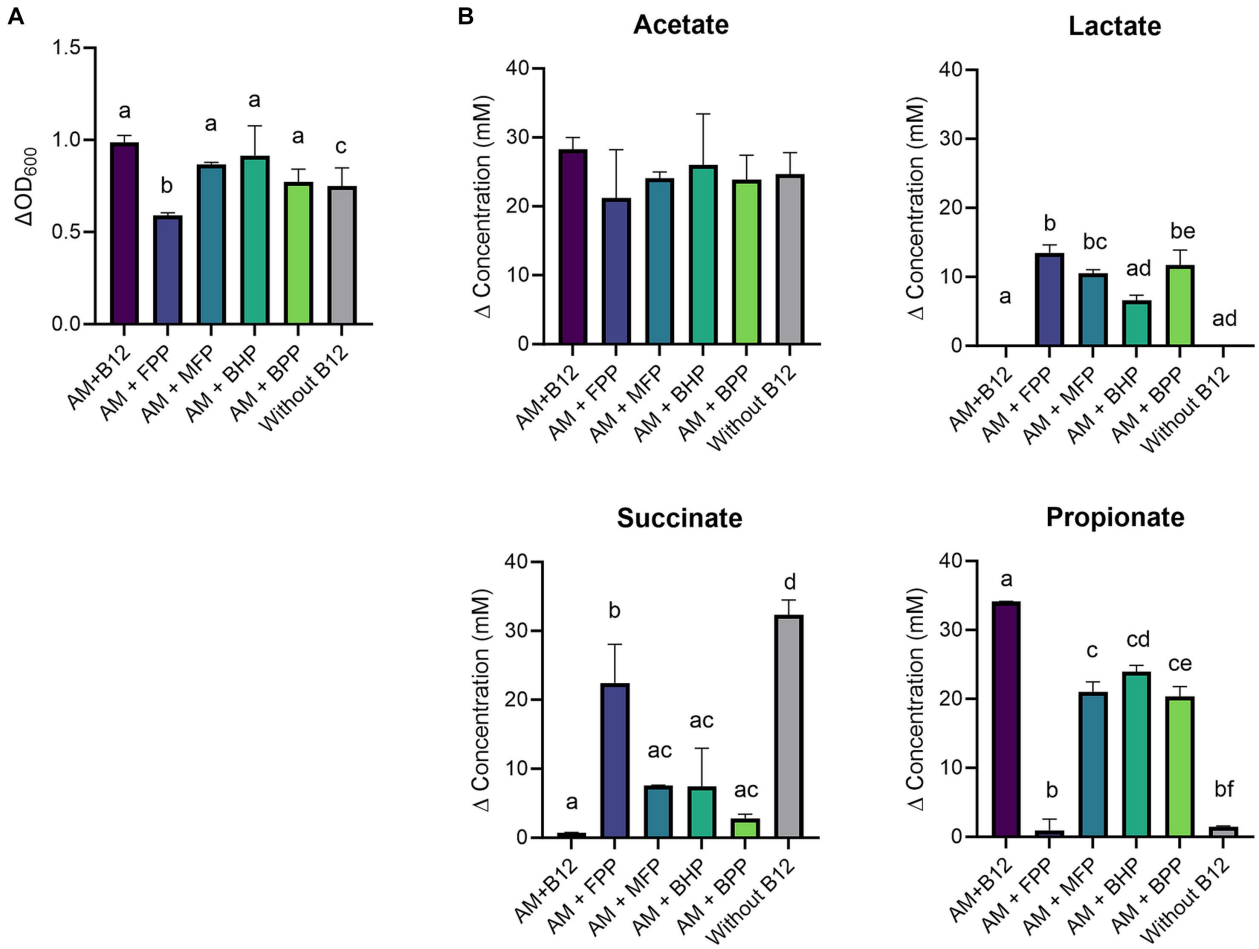
**(A)** Growth (ΔOD_600_) and **(B)** production (mM) of acetate, lactate, succinate, and propionate by *Akkermansia muciniphila* (AM) in basal medium with acetate (BMA) medium with B12 (as cyano-B12), without added B12, and in BMA with the inactivated bacterial (IB) preparations of the B12 prototrophic strains, *Blautia hydrogenotrophica* (BHP)*, Blautia producta* (BPP)*, Marvinbryantia formatexigens* (MFP) and of B12 non producer *Faecalibacterium prausnitzii* (FPP), after 72 h incubation at 37°C under anaerobic conditions. “P” stands for preparation. Average with standard deviation of three biological replicates is shown. Significant differences were calculated by one-way ANOVA, including Tukey’s test. Treatments with the same letter are not significantly different, while those with different letters are significantly different (*p* < 0.05).

*A. muciniphila* grew in all the tested conditions (Figure 1A). However, growth was significantly higher with added cyano-B12 (ΔOD_600_ 1.0 ± 0.1) when compared to the condition without B12 (ΔOD_600_ 0.7 ± 0.1). Similar growth was measured between IB preparations of *B. hydrogenotrophica* (BHP, ΔOD_600_ 0.9 ± 0.2), *M. formatexigens* (MFP, ΔOD_600_ 0.9 ± 0.01), *B. producta* (BPP, ΔOD_600_ 0.8 ± 0.1), but did not vary significantly when compared to the condition without B12. On the other hand, growth of *A. muciniphila* with the IB preparation of *F. prausnitzii* (FPP, ΔOD_600_ 0.6 ± 0.1) was significantly lower compared to the condition with added cyano-B12, BHP, MFP, and without B12.

When grown in medium without added B12, *A. muciniphila* produced the highest concentration of succinate (32.3 ± 2.6 mM) and low propionate (1.5 ± 0.9 mM) (Figure 1B). In contrast, an almost complete conversion of succinate (0.7 ± 0.1 mM) to propionate (34.1 ± 0.1 mM) was observed with added cyano-B12. With IB preparations containing pseudo-B12, conversion of succinate to propionate was also observed, but to a lesser extent compared to cyano-B12. For example, succinate was still detected in BPP (2.8 ± 0.6 mM), MFP (7.6 ± 0.1 mM) and BHP (7.5 ± 5.6 mM), but a notable amount of propionate was produced by *A. muciniphila* (BPP: 20.4 ± 1.4 mM, BHP: 23.9 ± 0.9 mM and MFP: 21.0 ± 1.4 mM). A significantly higher accumulation of succinate (22.5 ± 5.6 mM, *p* < 0.0001) and almost no production of propionate (less than 1 mM) was observed for *A. muciniphila* cultivated with FPP preparation. Acetate was produced in all the conditions (ranging from 21.2 ± 7.1 mM to 28.3 ± 1.8 mM), while lactate was only detected in the conditions when *A. muciniphila* was grown with IB preparations (ranged between 6.5 ± 0.7 mM to 11.7 ± 2.2 mM). Overall, these results suggest that B12 present in the IB preparations of the selected B12 prototrophs enhanced the growth of *A. muciniphila* and changed its metabolism, resulting in higher propionate production.

**Figure 2:**
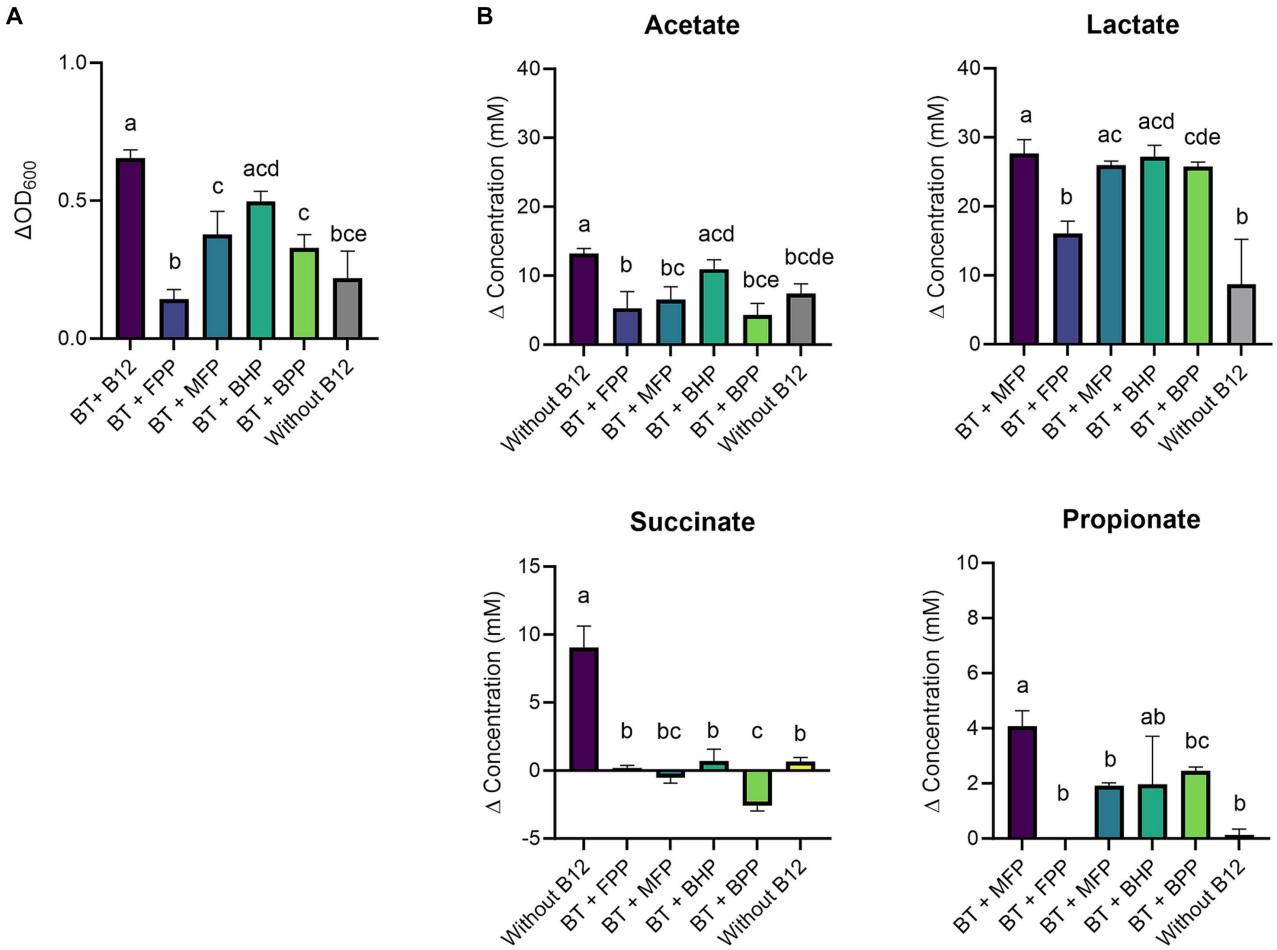
**(A)** Growth (ΔOD_600_) and **(B)** production (mM) of acetate, lactate, succinate, and propionate by *Bacteroides thetaiotaomicron* (BT) in basal medium with acetate (BMA) medium with B12 (as cyano-B12), without added B12, and in BMA with the inactivated bacterial (IB) preparations of the B12 prototrophic strains, *Blautia hydrogenotrophica* (BHP), *Blautia producta* (BPP), *Marvinbryantia formatexigens* (MFP) and of B12 non producer *Faecalibacterium prausnitzii* (FPP), after 72 h incubation at 37°C under anaerobic conditions. “P” stands for preparation. Average with standard deviation of three biological replicates is shown. Significant differences were calculated by one-way ANOVA, including Tukey’s test. Treatments with the same letter are not significantly different, while those with different letters are significantly different (*p* < 0.05).

The growth of *B. thetaiotaomicron* was significantly increased upon B12 supplementation when cyano-B12 (ΔOD_600_ 0.7 ± 0.03, *p* < 0.0001) and the IB preparation BHP (ΔOD_600_ 0.5 ± 0.03, *p* = 0.0012) were added, in comparison to medium without B12 (ΔOD_600_ 0.2 ± 0.1) and with the other IB preparation (Figure 2A). Overall, without added B12 (FPP and without B12), the growth of *B. thetaiotaomicron* was lowest and not all glucose was consumed, with 13.8 ± 1.4 mM and 20.1 ± 4.8 mM remaining from 27 mM glucose added in FPP and without B12 condition, respectively (Supplementary Figure S5).

At a metabolic level, *B. thetaiotaomicron* produced significantly more succinate (9.0 ± 1.6 mM, *p* < 0.0001) and propionate (4.1 ± 0.6 mM, *p* = 0.0004) with cyano-B12 compared to the conditions without added B12 (both succinate and propionate were less than 1 mM) (Figure 2B). Overall, propionate was only detected after growth in medium with IB preparations containing B12 (BHP: 1.9 ± 1.7 mM, BPP: 2.5 ± 0.2 mM, and MFP: 1.9 ± 0.1). On the other hand, acetate was produced in all conditions, ranging from 4.3 ± 1.7 mM to 13.2 ± 0.8 mM. Interestingly, high levels of lactate were detected in all the conditions, but they were significantly higher with cyano-B12 and the IB preparations containing pseudo-B12 of MFP, BHP and BPP (ranging between 25.0 to 27.0 mM), compared to the conditions without added B12 (no B12: 8.7 ± 6.5 mM and FPP: 16.1 ± 1.8 mM). To summarize, when cultivated in BMA medium, growth of *B. thetaiotaomicron* was promoted by B12, and the strain produced lactate rather than propionate.

**Figure 3:**
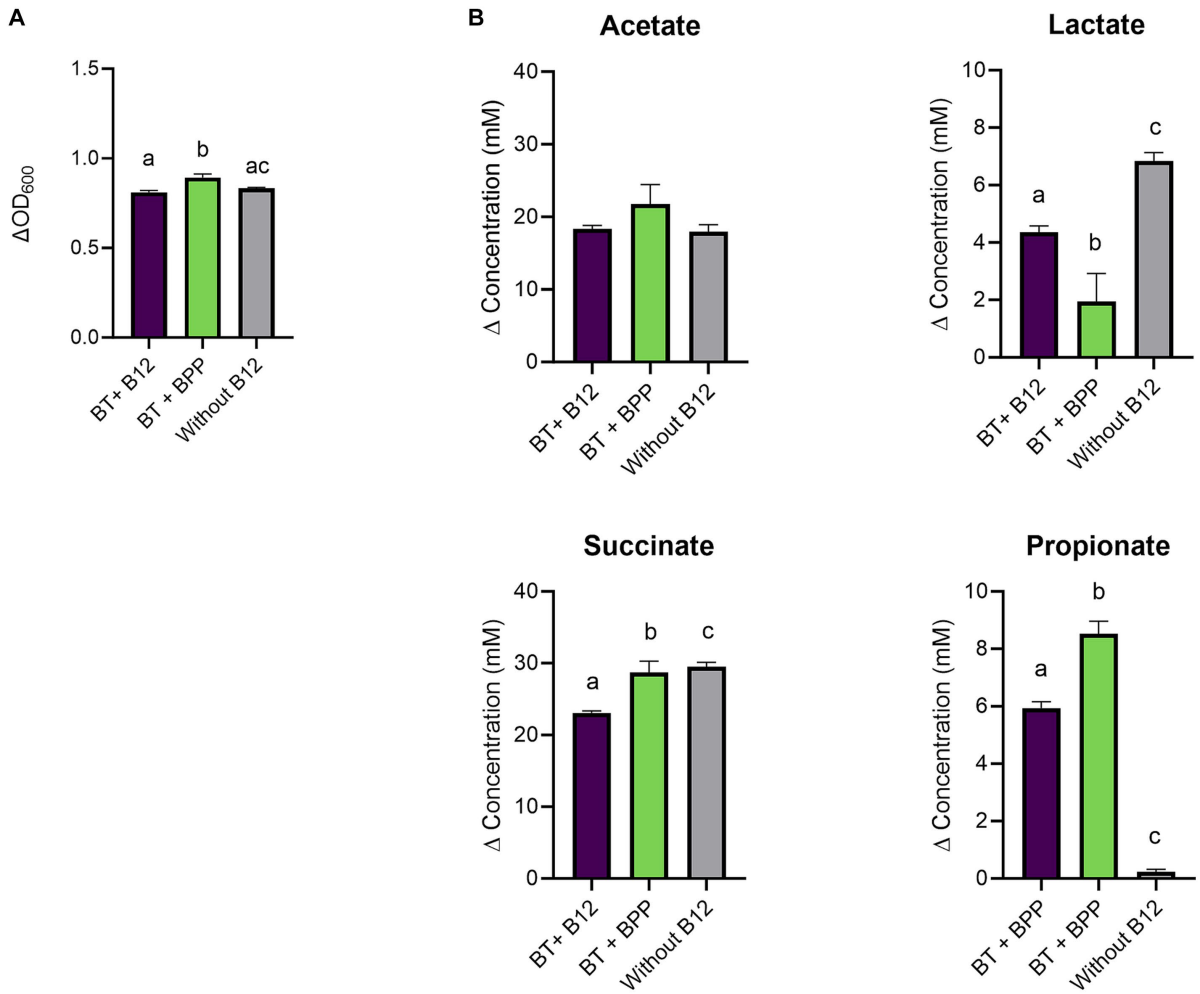
**(A)** Growth (ΔOD_600_) and **(B)** production (mM) of acetate, lactate, succinate, and propionate by *Bacteroides thetaiotaomicron* (BT) in chemically defined medium (CDM) medium with B12 (as cyano-B12), without added B12, and with the inactivated bacterial (IB) preparation of the B12 prototrophic strain, *Blautia producta*, BPP, after 72 h incubation at 37°C under anaerobic conditions. “P” stands for preparation. Average with standard deviation of three biological replicates is shown. Significant differences were calculated by one-way ANOVA, including Tukey’s test. Treatments with the same letter are not significantly different, while those with different letters are significantly different (*p* < 0.05).

Therefore, the experiment was repeated in a medium that allowed better assessment of the impact of cyano-B12 and pseudo-B12 on propionate production. It was hypothesized that the BMA medium selected for *B. thetaiotaomicron* was less suitable for the succinate pathway, possibly due to high concentrations of the nitrogen source in the medium (ca. 24 g/L). (40) *B. thetaiotaomicron* was then cultivated in chemically defined medium (CDM; with low nitrogen levels supplied in the form of vitamin-free casein hydrolysate (10 g/L) and mixture of amino acids (total 2 g/L)) with 10 ng cyano-B12/ml, without B12 and with IB preparation of *B. producta*, BPP (75.1 ng pseudo-B12/ml). Growth and SCFA were measured after 72 h incubation. *B. thetaiotaomicron* grew in all conditions (ranged between ΔOD_600_ 0.8 ± 0.01 to 0.9 ± 0.02; Figure 3A), with BPP showing significantly more growth compared to cyano-B12 (BPP: 0.9 ± 0.02, B12: 0.8 ± 0.01, *p* = 0.0008) and without B12 (0.8 ± 0.004, *p* = 0.0043). Regarding metabolite production in CDM, in the absence of B12, *B. thetaiotaomicron* accumulated the highest concentration of succinate (29.5 ± 0.6 mM) with no propionate produced (Figure 3B). For propionate production, BPP resulted in significantly more propionate (8.5 ± 0.4 mM, *p* < 0.0001) compared to the condition with B12 (5.9 ± 0.2 mM). For acetate, similar levels were produced in all the conditions, ranging from 17.96 ± 0.9 without B12 to 21.7 ± 2.6 mM in BPP. Indeed, lower levels of lactate compared to cultivation in BMA were observed in all the conditions (ranged between 1.9 to 6.8 mM). Although succinate was still detected in this medium, the experiment in CDM confirmed that microbially-produced B12 can be utilized by *B. thetaiotaomicron* for succinate to propionate conversion.

### 3.3 Higher doses of commercial B12 analogues increase propionate production by *Akkermansia muciniphila* and *Bacteroides thetaiotaomicron*

Next, the effect of different B12 analogues (cyano-B12, adenosyl-B12 and hydroxy-B12) that occur in the human diet and are available commercially was assessed. For this, growth and propionate production of *A. muciniphila* and *B. thetaiotaomicron* was assessed in BMA medium at two B12 analogue doses based on standard gut microbial cultivation media compositions, low (10 μg/L) and high (200 μg/L), reported as 1x and 20x, respectively.

**Figure 4:**
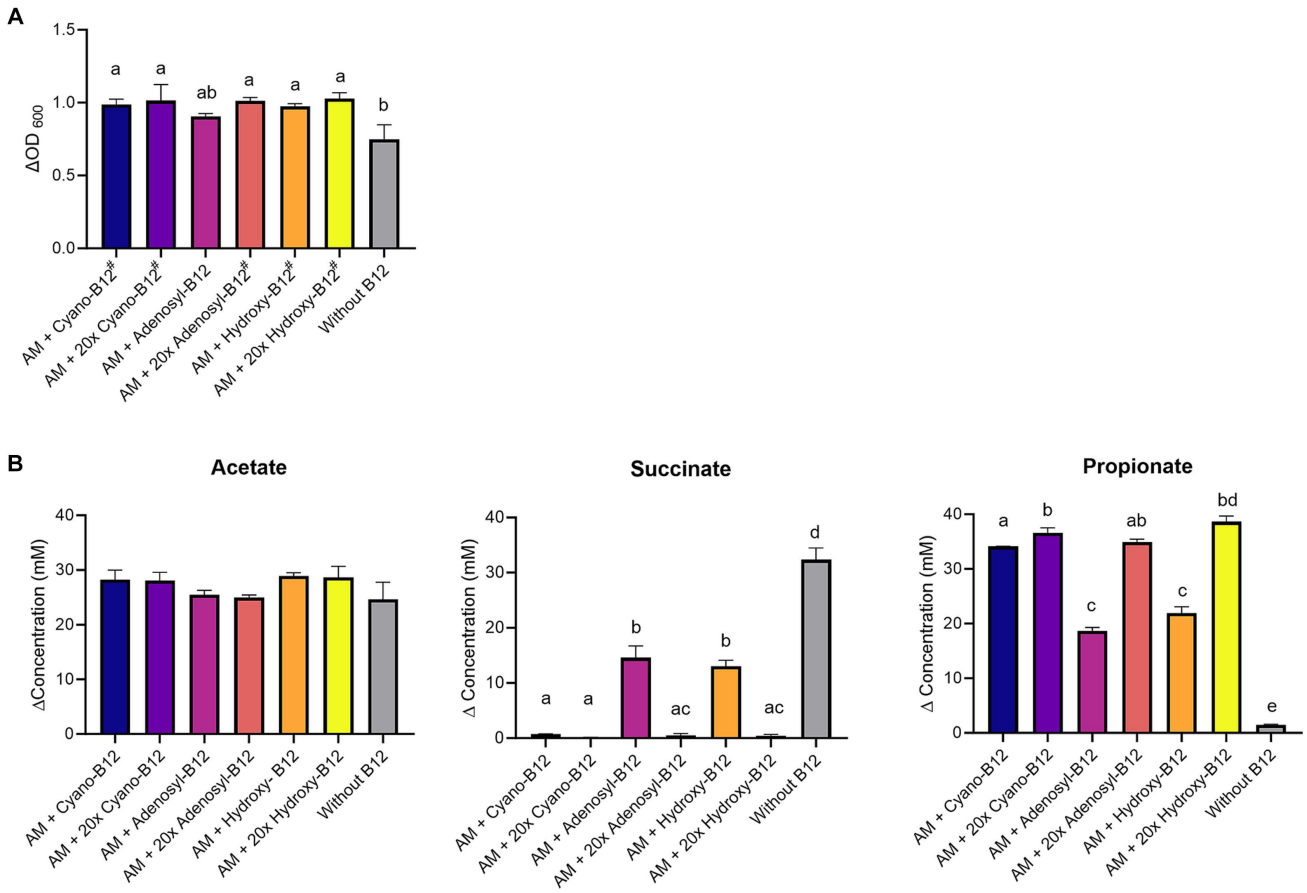
**(A)** Growth (ΔOD_600_) and **(B)** production (mM) of acetate, succinate, and propionate by *Akkermansia muciniphila* (AM) in basal medium with acetate (BMA) medium with B12 (shown as cyano B12), “20x” cyano-B12, adenosyl-B12, “20x” adenosyl-B12, hydroxy-B12, “20x” hydroxy-B12 and without added B12, after 72 h incubation at 37°C under anaerobic conditions. The “20x” refers to 20 times the concentration of the 1x concentration (10 μg/L). Average with standard deviation of three biological replicates is shown. Significant differences were calculated by one-way ANOVA, including Tukey’s test. Treatments with the same letter are not significantly different, while those with different letters are significantly different (*p* < 0.05). Lactate production was below detection limit (less than 1 mM).

*A. muciniphila* grew in all conditions, but the growth was significantly (*p* < 0.05) higher in the presence of all conditions with added B12 analogue, except for adenosyl-B12 (ΔOD_600_ 0.91 ± 0.02, *p* = 0.07), when compared to the medium without B12 (ΔOD_600_ 0.75 ± 0.1) (Figure 4A). However, growth did not vary significantly among B12 treatments (between cyano-B12 vs. adenosyl-B12 vs. hydroxy-B12) and between different doses.

On a metabolic level, a significantly higher succinate accumulation (32.3 ± 2.2 mM, *p* < 0.05) and lower propionate formation (1.5 ± 0.1 mM) was observed in conditions without added B12 when compared to all other conditions containing B12 (Figure 4B). Additionally, at 1x dose, only partial conversion of succinate to propionate was observed for adenosyl-B12 (succinate: 14.6 ± 2.4 mM and propionate: 18.7 ± 0.6 mM) and hydroxy-B12 (succinate: 13.0 ± 1.1 mM and propionate: 21.9 ± 1.2 mM) in comparison to cyano-B12 (succinate: 0.7 ± 0.1 mM and propionate: 34.1 ± 0.1 mM). Interestingly, the higher doses resulted in complete succinate conversion and high propionate formation (20x cyano-B12, 36.6 ± 0.9 mM; 20x adenosyl-B12, 34.9 ± 0.5 mM and 20x hydroxy B12, 38.7 ± 1.0 mM). Acetate was detected in all conditions (from 24.7 ± 3.1 mM to 28.9 ± 0.6 mM) but did not vary significantly between conditions and no lactate was detected in any tested conditions. Overall, our results show that higher doses of adenosyl-B12 and hydroxy-B12 induce a complete conversion of succinate to propionate by *A. muciniphila*.

**Figure 5:**
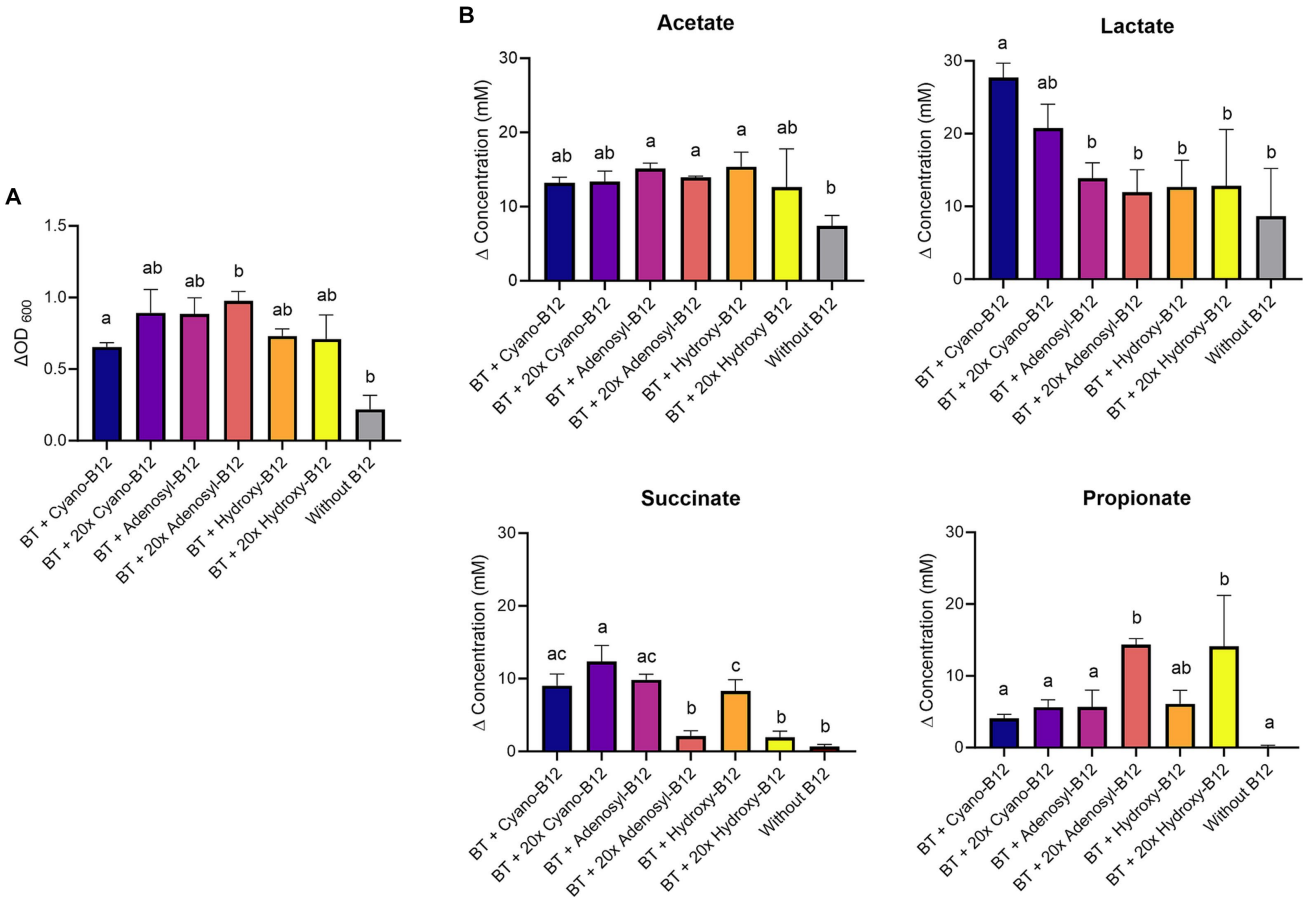
**(A)** Growth (ΔOD_600_) and **(B)** production (mM) of acetate, lactate, succinate, and propionate by *Bacteroides thetaiotaomicron* (BT) in basal medium with acetate (BMA) medium with B12 (shown as cyano-B12), “20x” cyano-B12, adenosyl-B12, “20x” adenosyl-B12, hydroxy-B12, “20x” hydroxy-B12 and without added B12, after 72 h incubation at 37°C under anaerobic conditions. The “20x” refers to 20 times the concentration of the 1x concentration (10 μg/L). Average with standard deviation of three biological replicates is shown. Significant differences were calculated by one-way ANOVA, including Tukey’s test. Treatments with the same letter are not significantly different, while those with different letters are significantly different (p < 0.05).

*B. thetaiotaomicron* grew in all conditions (Figure 5A), but the growth was significantly higher in conditions containing B12 (range between ΔOD_600_ 0.6 ± 0.03 to 1.0 ± 0.1) compared to the medium without B12 (ΔOD_600_ 0.2 ± 0.1). However, it did not vary significantly among the different B12 treatments.

In terms of metabolite production, no succinate or propionate production was observed in the absence of B12. *B. thetaiotaomicron* produced significantly more propionate with 20x adenosyl-B12 (14.3 ± 0.9 mM, *p* = 0.03) compared to 20x cyano-B12 (5.6 ± 1.0 mM). *B. thetaiotaomicron* accumulated less succinate and produced more propionate with 20x adenosyl-B12 and 20x hydroxy-B12, compared to growth with lower doses (Figure 5B). Acetate was produced in all conditions but was higher with B12 (ranging between 12.6 ± 5.1 mM to 15.4 ± 1.9 mM) than without B12 (7.4 ± 1.4 mM). Moreover, high lactate production was evident in all the conditions, but was significantly higher in conditions with cyano-B12 (27.6 ± 1.9 mM), compared to without B12. Interestingly, lactate concentration was significantly lower in the conditions with both low and high doses of adenosyl-B12 (13.8 ± 2.1 mM and 11.9 ± 3.1 mM, respectively) and hydroxy-B12 (12.7 ± 3.7 mM and 12.8 ± 7.7 mM, respectively), when compared to cyano-B12.

**Figure 6:**
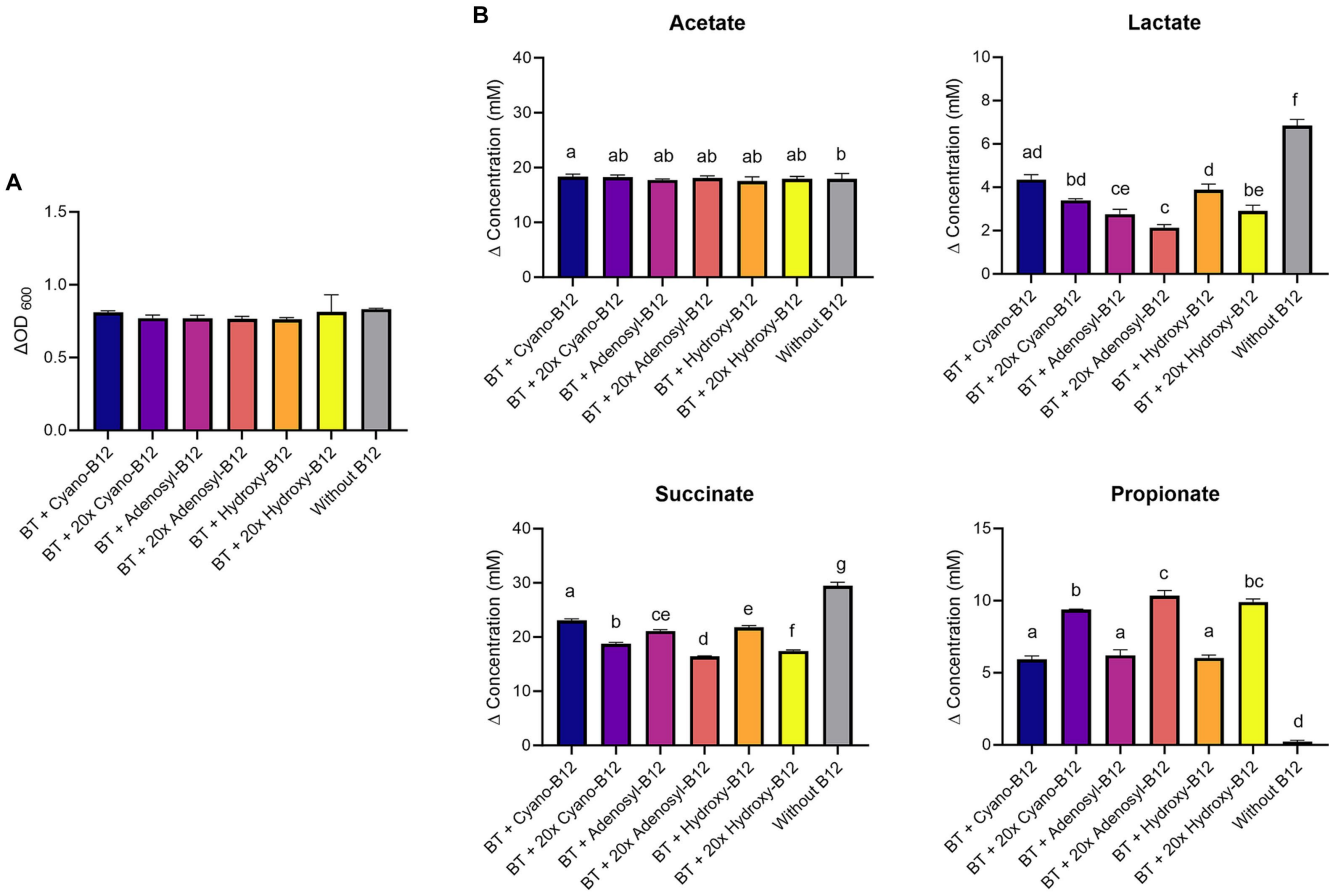
**(A)** Growth (ΔOD_600_) and **(B)** production (mM) of acetate, lactate, succinate, and propionate by *Bacteroides thetaiotaomicron* (BT) in chemically defined medium (CDM) medium with B12 (shown as cyano-B12), “20x” cyano-B12, adenosyl-B12, “20x” adenosyl-B12, hydroxy-B12, “20x” hydroxy-B12 and without B12 after 72 h incubation at 37°C under anaerobic conditions. The “20x” refers to 20 times the concentration of the 1x concentration (10 μg/L). Average with standard deviation of three biological replicates is shown. Significant differences were calculated by one-way ANOVA, including Tukey’s test. Treatments with the same letter are not significantly different, while those with different letters are significantly different (*p* < 0.05).

Because of the high lactate production detected in BMA medium, the impact of different B12 analogues and doses on propionate metabolism of *B. thetaiotaomicron* was again evaluated with CDM. *B. thetaiotaomicron* showed the same growth in all conditions in CDM (ranged from ΔOD_600_ 0.8 ± 0.1 to 0.9 ± 0.02) (Figure 6A). Lower levels of lactate were detected in all the conditions with B12 (ranged between 1.7 to 4.3 mM), compared to no B12. Conditions containing the 1x dose resulted in higher succinate accumulation (ranged between 21.1 and 23.1 mM) and lower propionate production (ranged between 5.9 and 6.2 mM), compared to the high dose (17.4 to 18.7 mM) and (9.3 to 10.4 mM), respectively (Figure 6B). For acetate, similar levels were produced in all conditions (17 to 18 mM). Overall, results in CDM confirmed that higher doses of cyano-B12, adenosyl-B12 and hydroxy-B12 enhance the propionate metabolism in the succinate pathway for *B. thetaiotaomicron.*

## 4 Discussion

In this study, the actual B12 production was demonstrated by 6 of the 8 B12-producing strains predicted *in silico* based on previous genome analysis studies (14, 25, 26). Their B12 production was quantified, which adds to the previous studies that detected but not quantified B12 production for gut bacterial strains, including *Eubacterium hallii*, *Eubacterium limosum* DSM 20517, *B. hydrogenotrophica*, and *Veillonella parvula* DSM 2008 (14, 18, 41, 42). The B12-producing strains had widely different B12 specific yields, expressed per 10^9^ cells to adjust for growth, with different intra- and extracellular distribution of B12 and pseudo-B12. For the first time it was demonstrated that prevalent gut microbes, such as *B. producta*, *M. formatexigens,* and *R. gauvreauii*, produce pseudo-B12 as the major form. Previously, Belzer et al. (15) also identified pseudo-B12 as the major B12 analogue produced by *E. hallii*. Our *in vitro* data suggest that pseudo-B12 is the primary B12 analogue produced in the gut, which is consistent with the high levels of pseudo-B12 detected in adult feces; pseudo-B12 represents approximately 79% of total B12 (21). The latter work by Allen and Stabler (2008) provides valuable insights into the distribution of corrinoids in the human gut and showed that 2-methyl pseudo-B12 was detected as the dominant form of B12 in the human feces. It is essential to note that our analytical method for detection and quantification of B12 did not allow us to distinguish between different forms of pseudo-B12 with various groups attached and thus our data present the overall pseudo-B12 production by gut microbes. Moreover, a recent comparative study by Hallberg et al. (43) suggests that 2-methyl pseudo-B12 is the primary cobamide in mammalian gut environments but with varying levels between feces and other regions of the gastrointestinal tract (43). For example, lower levels of 2-methyl pseudo-B12 were detected in the bovine duodenum compared to bovine rumen and bovine feces. Our interpretation aligns with the notion that higher pseudo-B12 levels in feces are a consequence of microbial production in the colon, where microbial load is higher compared to the duodenum.

Further, the high fraction of extracellular B12 in *R. gauvreauii* and *B. producta* implies that microbially-produced B12 can be released during fermentation, possibly due to cell lysis or to a not yet identified export mechanism. Although *in situ* data are lacking, microbial cell lysis is a likely mechanism in the contribution of human gut microbiota to nutrient exchange among taxa, supported by the high fraction of permeable cell populations detected in fresh human feces (average 38% of total cells) (44). Therefore, B12 produced *in situ* may become available for cross-feeding to the auxotrophic members of the gut microbial community.

Next, it was shown that the presence of B12 analogues produced by gut microbes or derived from commercially available B12 forms promoted the growth of two common B12 auxotrophic propionate-producing gut bacteria, *A. muciniphila* and *B. thetaiotaomicron*. This may explain the findings of a study in healthy men reporting that high dietary B12 consumption was associated with the higher relative abundance of *Akkermansia* in colonic mucus-associated microbiota, compared to low dietary B12 consumption (45). *B. thetaiotaomicron* is known to employ BtuG2 (a lipoprotein) to bind B12 so tightly that it can even scavenge B12 from human intrinsic factor, a key B12 transport protein in humans (46). *B. thetaiotaomicron* and *A. muciniphila* depend on methionine for their growth, which is synthesized by B12 dependent MetH enzyme and can explain the growth-promoting effect of B12 (27, 28). Basal medium with acetate (BMA) contained tryptone as methionine-source and its concentration was estimated 10x less (0.021 g/L based on technical info on Bacto Tryptone (39)) than CDM (0.29 g/L). This potential difference in methionine availability between the two media could explain why no decreased growth was observed for *B. thetaiotaomicron* when cultivated in CDM without added B12.

The B12 produced by *B. hydrogenotrophica*, *M. formatexigens* and *B. producta* promoted succinate to propionate conversion by *A. muciniphila*. This is in line with a previous *in vitro* study where pseudo-B12 produced by *Eubacterium hallii* L2-7 promoted propionate production by *A. muciniphila* (15). *A. muciniphila* possesses the B12 remodeling enzyme CbiR, which transfers available B12 to its preferred analogue, pseudo-B12 (27). Of note, pseudo-B12 analogues have low biological activity in humans since they cannot function as cofactors for methionine synthase and methylmalonyl-CoA mutase (20, 22).

In contrast, with *B. thetaiotaomicron,* only partial conversion of succinate to propionate was noted with all tested B12 analogues. This led us to speculate that B12 analogues may be redirected for other vital functions within the metabolic processes of *B. thetaiotaomicron*. The presence of multiple B12 transporters on its surface, capable of binding different B12 analogues, suggests that they play a crucial and multifaceted role in metabolism of *B. thetaiotaomicron* (28).

Furthermore, it is noteworthy that propionate production with IB preparations was comparatively lower in *A. muciniphila* and *B. thetaiotaomicron* when compared to the condition with added cyano-B12. This observation is intriguing, especially considering that all three IB preparations contained higher levels of detected B12 than the condition with added B12 (10 ng/mL). These observations can potentially be attributed to the stability of the cyano-B12 form (23).

This study showed that in contrast to cyano-B12, higher doses of adenosyl-B12 and hydroxy-B12 promoted conversion of succinate to propionate in *B. thetaiotaomicron*; this finding requires further experimentation to deduce a possible biological mechanism. Lower succinate levels and higher propionate levels in the gut are expected to have a beneficial impact on host health, considering that succinate accumulation is widely reported in diseases such as inflammatory bowel disease (IBD) (47, 48). Lower gut B12 levels are expected in IBD patients, as previous studies showed a decreased microbial B12 synthesis and lower B12 fecal levels (49–51). Interestingly, there is a substantial reduction in the abundance of propionate-producing species, specifically *A. muciniphila*, in IBD, and especially in patients with Crohn’s disease below the age of 16 (52, 53). Our finding emphasizes the need for additional investigation, which could pave the way for promising future research aimed at developing potential therapeutic interventions for conditions like IBD.

To conclude, our study provides valuable insights into the *in vitro* production of B12 by gut microbial strains with pseudo-B12 being the dominant analogue. For the first time it was shown that different B12 analogues impact propionate production by the common propionate-producing gut microbes, *A.muciniphila* and *B. thetaiotaomicron*, by converting succinate to propionate. Additionally, different analogues of commercially available B12 (cyano-B12, adenosyl-B12 and hydroxy-B12) display a dose- dependent effect, with higher doses promoting succinate to propionate conversion. However, such observations could be dependent on the environmental growth conditions, as shown for *B.thetaiotaomicron* metabolism and the observed effect of the nitrogen source. By using propionate-producing single strains, it was confirmed that high doses of B12 can increase their propionate production as seen previously in a fecal batch incubation study (16). However, further studies are needed to understand how low and high doses of different B12 analogues affect propionate production in complex gut communities, and their underlying biological mechanisms and implications of these findings in the context of gut microbiota-mediated health outcomes. Overall, this study contributes to the growing body of knowledge on B12 analogue production by gut microbes and its potential to promote propionate production in the gut with possible implication for human gut health.

## Data availability statement

The datasets presented in this study can be found in online repositories. The names of the repository/repositories and accession number(s) can be found at: https://doi.org/10.3929/ethz-b-000638554.

## Author contributions

PK: Conceptualization, Data curation, Formal analysis, Investigation, Methodology, Visualization, Writing – original draft, Writing – review & editing. AGr: Conceptualization, Funding acquisition, Writing – review & editing. MD: Investigation, Writing – review & editing. SP: Investigation, Writing – review & editing. BP: Formal analysis, Investigation, Methodology, Writing – review & editing. CL: Conceptualization, Funding acquisition, Resources, Supervision, Writing – review & editing. AGe: Conceptualization, Funding acquisition, Investigation, Supervision, Writing – review & editing.
