# Supplementary material for: Vitamin B12 analogues from gut microbes and diet differentially impact commensal propionate producers of the human gut
Source: Front Nutr. 2024 Feb 8;11:1360199. doi: 10.3389/fnut.2024.1360199 (PMC10881866; doi:10.3389/fnut.2024.1360199)
Supplement: Supplementary file 1 [file Data_Sheet_1.docx]

Supplementary Material

**Supplementary Table S1:** B12 content measured by UHPLC-DAD in IB preparations of the selected strains. Limit of detection (30 ng/ml), ND: not detected. Only pseudo-B12 is detected. Data present values from three technical replicates.

| **Experimental strains** | **B12 content (ng/ml)** | **Log total bacteria (cells/ml)** |
| --- | --- | --- |
| *Blautia producta* DSM 14466 | 150.2 ± 3.8 | 8.6 |
| *Blautia hydrogenotrophica* DSM 10507 | 88.4 ± 1.9 | 9.6 |
| *Marvinbryantia formatexigens* DSM 14469 | 130.6 ± 2.2 | 8.9 |
| *Faecalibacterium prausnitzii* A2-165 | ND | 8.9 |

**
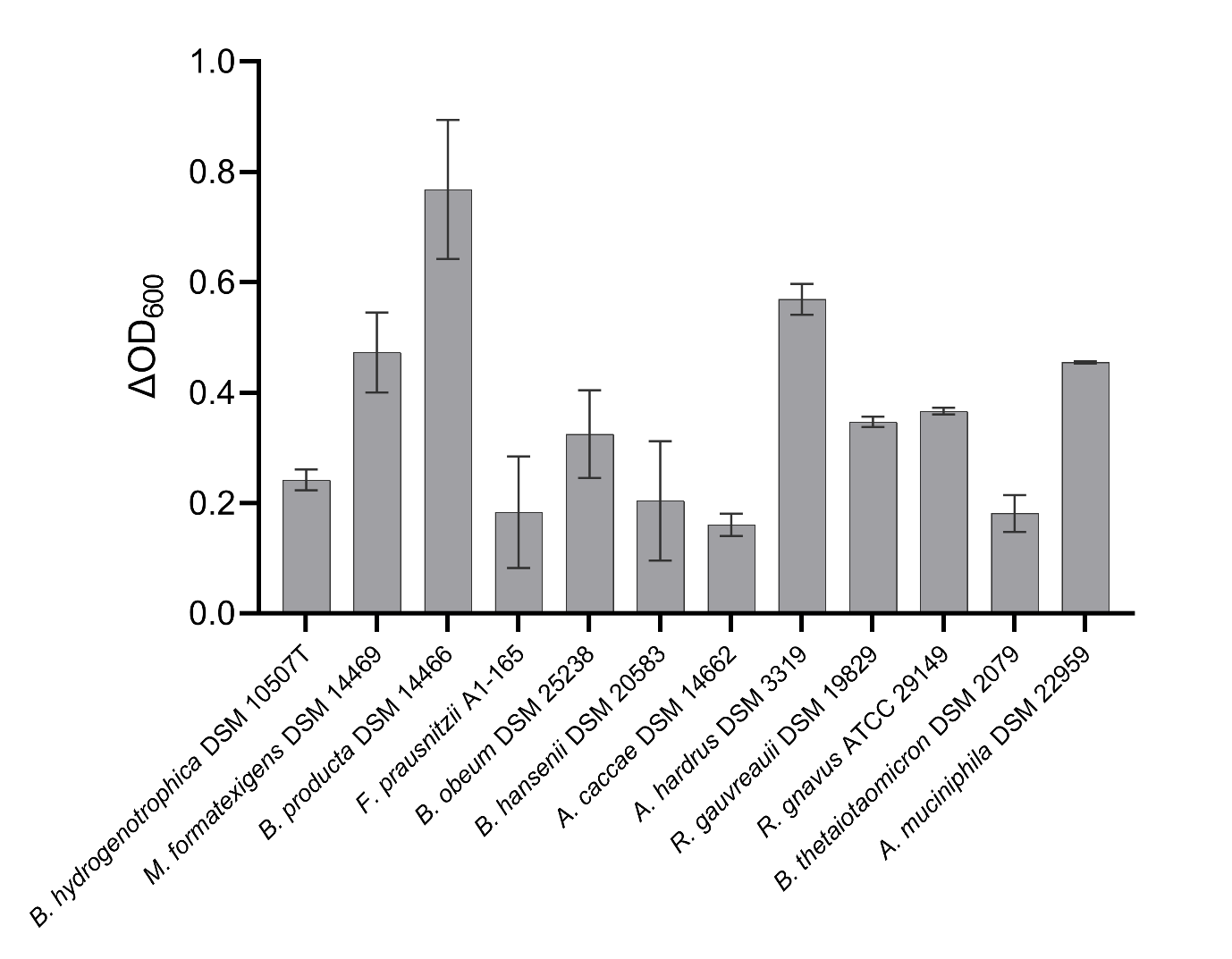
**

**Supplementary Figure S1:** Growth (ΔOD_600_) of gut bacterial strains during batch incubation in respective medium without added B12 after 48 h. ΔOD_600_ refers to the difference between the measured OD_600_ after 48 h and to OD_600_ of the blank media of each condition. Data represents average and standard deviation of three biological replicates.

**
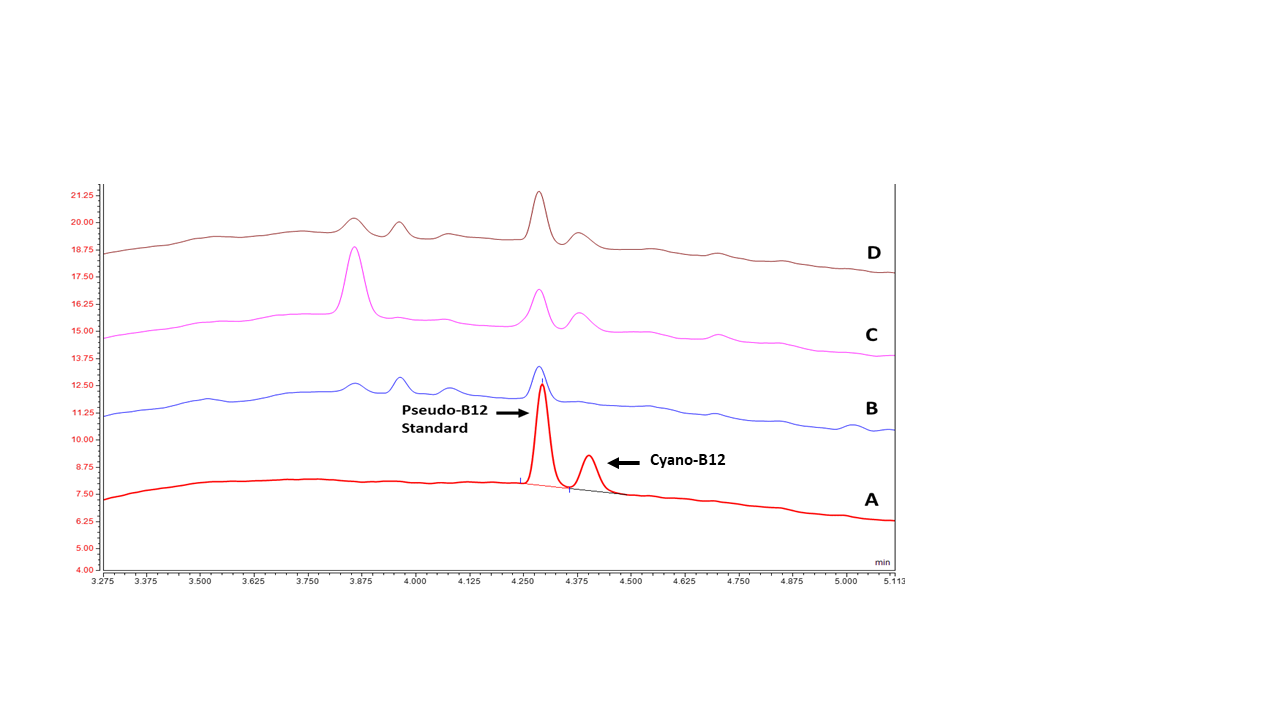
**

**Supplementary Figure S2:** UHPLC-DAD chromatograms of the **A)** pseudo-B12 and cyano-B12 standards, and of the intracellular samples of **B)** *B. producta*, **C)** *B. hydrogenotrophica*, and **D)** *M. formatexigens* at 360 nm wavelength. The strains were grown for 48 h in Basal medium supplemented with acetate (*B. producta*) and formate (*B. hydrogenotrophica* and *M. formatexigens*).

**
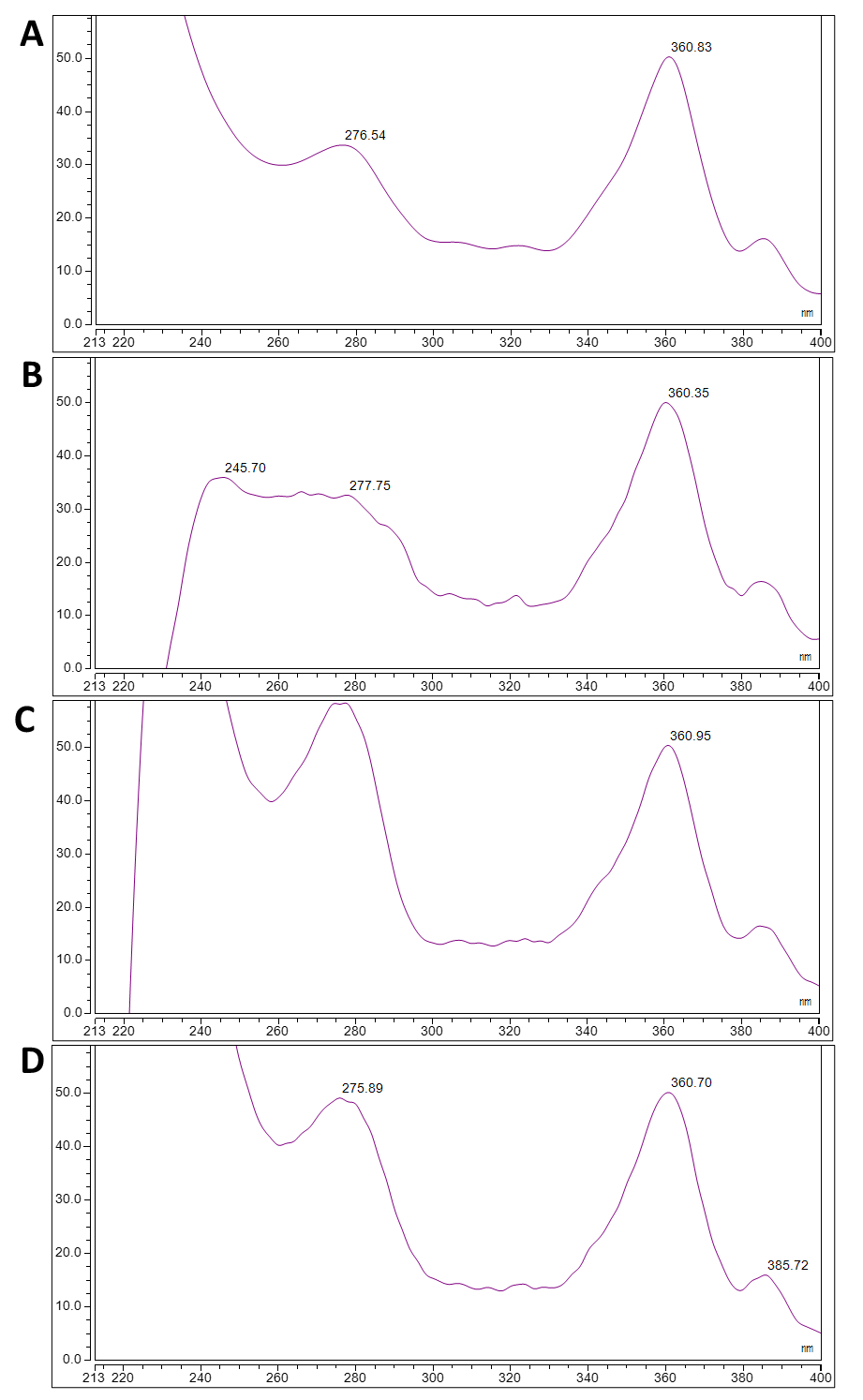
**

**Supplementary Figure S3**: UV absorption spectra of the **A)** pseudo-B12 standard, and the intracellular samples of **B)** *B. producta*, **C)** *B. hydrogenotrophica*, and **D)** *M. formatexigens*, indicating maximum absorption at 360 nm.

**
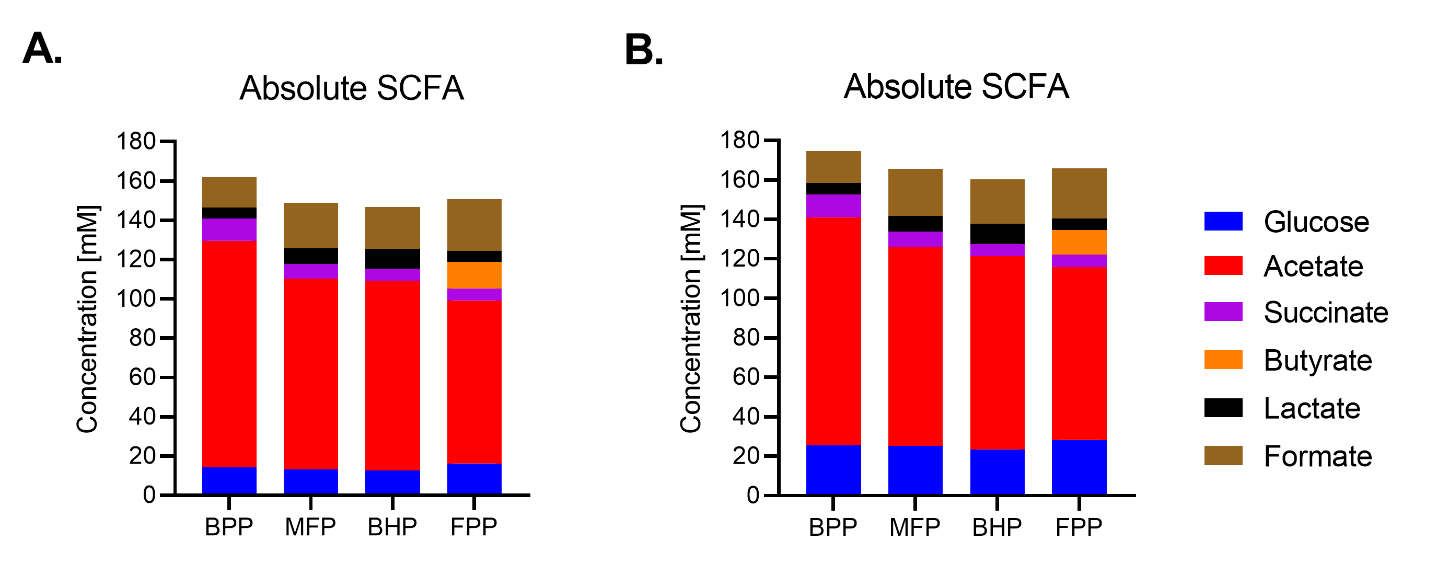
**

**Supplementary Figure S4:** Concentration (mM) of SCFA in BMA medium supplemented with IB preparations of B12 producing strains (*B. producta* BPP; *B. hydrogenotrophica*, BHP and *M. formatexigens, MFP*) and a non-producing strain (*F. prausnitzii*, FPP) used for (**A**) *A. muciniphila* and (**B**) *B. thetaiotaomicron*.


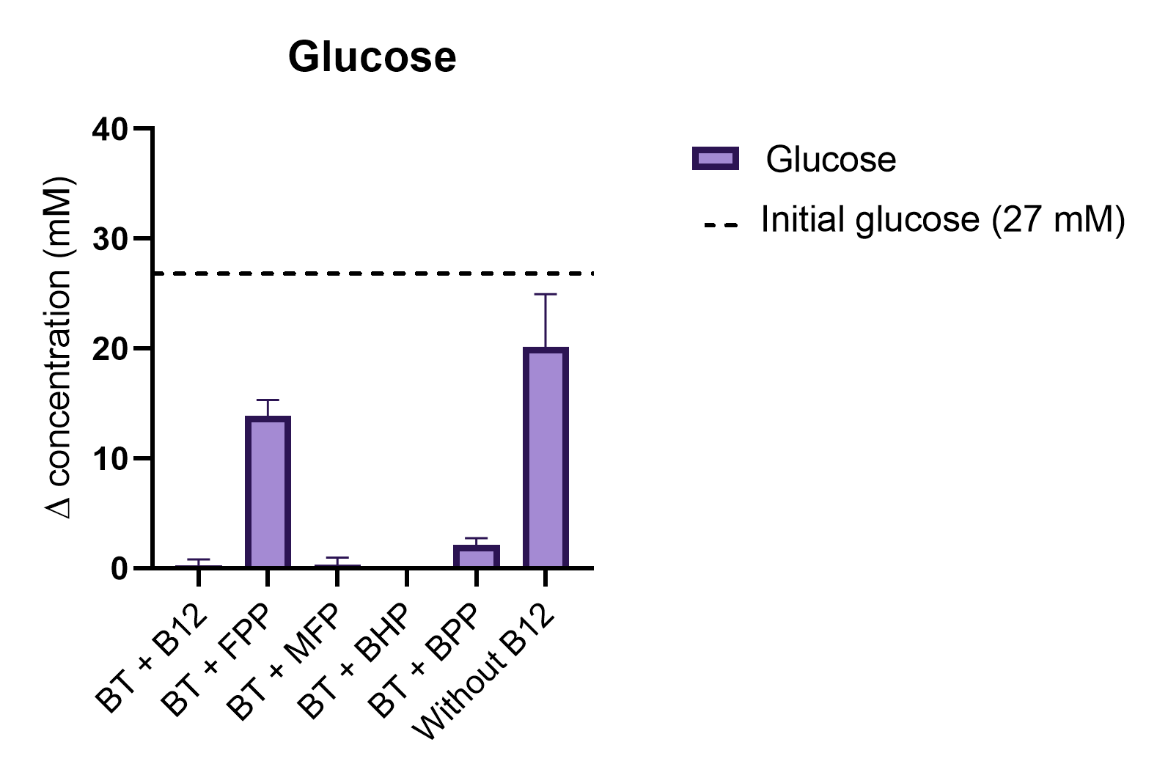


**Supplementary Figure S5:** Concentration of glucose remaining after 72 h of incubation of *B. thetaiotaomicron* (BT) in all conditions. *B. producta,* BPP; *B. hydrogenotrophica*, BHP; *M. formatexigens, MFP* and *F. prausnitzii*, FPP. “P” stands for preparation.
